# General Therapeutics and Meteria Medica

**Published:** 1854-01

**Authors:** 


*General Therapeutics and Materia Medica.* By Robley Dunglison, M. D., etc., fifth edition, revised and improved. Philadelphia: Blanchard & Lea, 1854.

THE *pharmacological* portions of the present edition have been thoroughly revised. The size of the volumes is increased. It is printed on good paper and is handsomely bound.

For sale by Keen & Bro's, Chicago. J.

